# Clinical Predictors for Delayed or Inappropriate Initial Diagnosis of Type A Acute Aortic Dissection in the Emergency Room

**DOI:** 10.1371/journal.pone.0141929

**Published:** 2015-11-11

**Authors:** Kazuhito Hirata, Minoru Wake, Takanori Takahashi, Jun Nakazato, Nobuhito Yagi, Tadayoshi Miyagi, Junichi Shimotakahara, Hidemitsu Mototake, Toshiho Tengan, Tsuyoshi R. Takara, Yutaka Yamaguchi

**Affiliations:** 1 Division of Cardiology, Okinawa Chubu Hospital, 281 Miyasato, Uruma, Okinawa, Japan; 2 Division of Cardiovascular Surgery, Okinawa Chubu Hospital, 281 Miyasato, Uruma, Okinawa, Japan; 3 Division of Emergency Department, Okinawa Chubu Hospital, 281 Miyasato, Uruma, Okinawa, Japan; Azienda Ospedaliero-Universitaria Careggi, ITALY

## Abstract

**Background:**

Initial diagnosis of acute aortic dissection (AAD) in the emergency room (ER) is sometimes difficult or delayed. The aim of this study is to define clinical predictors related to inappropriate or delayed diagnosis of Stanford type A AAD.

**Methods:**

We conducted a retrospective analysis of 127 consecutive patients with type A AAD who presented to the ER within 12 h of symptom onset (age: 69.0 ± 15.4 years, male/female = 49/78). An inappropriate initial diagnosis (IID) was considered if AAD was not included in the differential diagnosis or if chest computed tomography or echocardiography was not performed as initial imaging tests. Clinical variables were compared between IID and appropriate diagnosis group. The time to final diagnosis (TFD) was also evaluated. Delayed diagnosis (DD) was defined as TFD > third quartile. Clinical factors predicting DD were evaluated in comparison with early diagnosis (defined as TFD within the third quartile). In addition, TFD was compared with respect to each clinical variable using a rank sum test.

**Results:**

An IID was determined for 37% of patients. Walk-in (WI) visit to the ER [odds ratio (OR) 2.6, 95% confidence interval (CI) = 1.01–6.72, P = 0.048] and coronary malperfusion (CM, OR = 6.48, 95% CI = 1.14–36.82, P = 0.035) were predictors for IID. Overall, the median TFD was 1.5 h (first/third quartiles = 0.5/4.0 h). DD (>4.5 h) was observed in 27 cases (21.3%). TFD was significantly longer in WI patients (median and first/third quartiles = 1.0 and 0.5/2.85 h for the ambulance group vs. 3.0 and 1.0/8.0 h for the WI group, respectively; P = 0.003). Multivariate analysis revealed that WI visit was the only predictor for DD (OR = 3.72, 95% CI = 1.39–9.9, P = 0.009). TFD was significantly shorter for appropriate diagnoses than for IIDs (1.0 vs. 6.0 h, respectively; P < 0.0001).

**Conclusions:**

WI visit to the ER and CM were predictors for IID, and WI was the only predictor for DD in acute type A AAD in the community hospital.

## Introduction

Acute aortic dissection (AAD) is a life-threatening emergency that requires quick and accurate diagnosis as a delay in treatment carries a high mortality rate [1–4]. However, despite recent advances in medical diagnostic technology, quick and accurate diagnosis of AAD can be difficult because of the wide variety of clinical presentations and complications associated with this diagnosis [2–7]. Patients with AAD are often misdiagnosed, for example, with acute coronary syndrome (ACS), gastrointestinal diseases, and cerebrovascular accidents (CVA) [5–11]. Currently, risk factors for inappropriate initial diagnosis (IID) or delayed diagnosis (DD) of AAD have not been well defined. This study was conducted to identify clinical predictors that may contribute to IID or DD of acute type A AAD in order to improve mortality/morbidity in this patient cohort.

## Materials and Methods

From 1983 to 2011, of the 417 patients with AAD admitted to Okinawa Chubu Hospital, 227 presented directly to the emergency room (ER), without referral from another hospital, within 12 h from the onset of symptoms. Of these 227 patients, 127 with type A AAD were included in this retrospective analysis. The remaining 100 patients had type B AAD. The diagnosis of AAD was based on computed tomography (CT), echocardiography, angiography, or autopsy findings. Detailed history, vital signs, electrocardiogram (ECG), chest roentgenogram (chest X-ray), and CT findings were retrospectively reviewed, and complications related to AAD were carefully evaluated. Cardiac tamponade was considered present if hypotension and/or evidence of cardiac compression were present with pericardial effusion. The diagnosis of aortic regurgitation was based on auscultation and color-Doppler echocardiography (or aortography in the early 1980s). Peripheral artery occlusion (PAO) was considered present if there was a pulse deficit or absent contrast enhancement with CT. Kidney and visceral ischemia was defined by the absence of contrast enhancement with CT. Congestive heart failure (CHF) was defined by symptoms of dyspnea associated with chest X-ray findings suggestive of CHF. Coronary malperfusion was defined based on direct visualization by the cardiac surgeon at the time of surgery or an ECG finding of segmental ST elevation consistent with the distribution of the coronary artery. Acute ECG changes were defined as a shift in ST segment ≥0.1 mV or a change in the polarity or the morphology of the T wave (inversion of the previously normal T wave or vice versa), compared with previous or later ECGs. Preexisting changes, such as left ventricular hypertrophy, bundle branch block, and abnormal Q wave were considered as chronic changes. The type of dissection was defined according to CT, surgical, or autopsy findings. This research was approved by the Institutional Review Board of Okinawa Chubu Hospital, and the data were anonymously analyzed.

Initial management in the ER was mainly performed by resident physicians (postgraduate year 2 or 3) supervised by ER staff doctors. Doctors were encouraged to describe possible differential diagnoses. If the detailed information was not described in the chart, doctors who initially took care of the patients were contacted and detailed information regarding their initial differential diagnosis was collected.

Two different analytic approaches were used. The first approach was to divide patients into two groups: those with an appropriate initial diagnosis (AID) and those with IID. The IID group comprised those patients for whom AAD was not included in the initial differential diagnoses as well as those patients for whom CT of the chest or a focused echocardiogram at the bedside were not performed as the initial diagnostic imaging test. Even when AAD was included in the initial differential diagnosis, imaging tests, such as brain CT, for possible CVA or abdominal CT or echography for possible acute abdomen were sometimes performed as initial imaging tests. In such cases, the initial diagnosis was regarded as inappropriate even if those imaging tests resulted in unexpected findings of AAD, assuming that AAD was not a top priority of the differential diagnosis. Clinical variables were compared between AID and IID groups using Student’s t-test for numerical data and Fisher’s exact test for categorical data. Logistic regression analysis was used for multivariate analysis for categorical data.

The second approach was to analyze the time required from admission to the ER to final diagnosis (time to final diagnosis: TFD) to identify any predictors of DD. TFD for each patient was calculated in 30-min intervals and expressed as median and first quartile and third quartile values. TFD was evaluated with respect to each clinical variable and was compared between variable-positive and -negative patients using a rank sum test. In addition, patients were divided into two groups according to TFD. The early diagnosis (ED) group included those with TFD shorter than the third quartile and the DD group included those with a TFD longer than the third quartile. Clinical variables were compared between ED and DD groups using Student’s t-test for numerical data and Fisher’s exact test for univariate analysis of categorical data. Multivariate analysis was performed using logistic regression tests for categorical data. A P value of <0.05 was considered statistically significant.

## Results

Table 1 shows patient demographics, clinical presentations, clinical complications, chest X-ray and ECG findings, and type of dissection. The mean age of the patients was 69.0 ± 15.4 years, and the male to female ratio was 1:1.6 with 49 males and 78 females. Hypertension was the most prevalent underlying condition, present in 67.7% of the patients. Pain was the main presenting symptom in 64.6% of the patients, with 78% of these patients experiencing sudden onset of pain. However, obtaining a detailed history regarding pain was occasionally difficult because 40% of the patients presented to the ER with an altered mental state. Approximately 9% of patients denied having any pain (true pain free). The mean initial systolic blood pressure (SBP) was 105 mmHg; 10% of the patients had an initial SBP > 160 mmHg and 48% of patients presented with shock, i.e., SBP < 90 mmHg. Despite the low blood pressure, initial heart rate was not very elevated (76.2 ± 20.9 beats/min). Regarding the mode of arrival, 78.8% of patients arrived by ambulance, whereas 21.3% walked in (WI). Furthermore, 80% of the patients had some type of complication related to AAD. The most common was cardiac tamponade, followed by aortic regurgitation and PAO. Coronary malperfusion due to dissection was observed in 5.5% of the patients. Widening of the mediastinum and cardiomegaly were the most common findings on chest X-ray followed by CHF (radiographic CHF). The incidence of abnormal ECG was as high as 77.2%, and ECG was normal in only 22% patients. Chronic abnormalities, such as left ventricular hypertrophy or bundle branch block as well as acute ST depression and/or T wave inversion were common ECG findings in the acute phase of AAD. Chest CT scan was obtained in 117 of 127 patients and was diagnostic in 111 (sensitivity of 94.9%). Bedside transthoracic echocardiogram was obtained in 104 patients, and an intimal flap was discovered in 57 (54.9%). Combining the results of these two tests led to a final diagnosis of AAD in 121 of 127 patients (sensitivity of 95.3%). In the remaining six patients, a diagnosis of AAD was made by aortography in three and by autopsy, transesophageal echocardiography, and surgery in one patient each. Ninety patients (70.9%) had classic dissection as manifested by an intimal flap, whereas 37 patients (29.1%) had intramural hematoma. Eighty-two patients underwent surgery with an in-hospital mortality rate of 17.1% (14 of 82), whereas 45 patients were treated medically with a mortality rate of 67% (30 of 45). The overall in-hospital mortality was 34.6% (44 of 127) in the present study. In 80 patients (63.0%), initial diagnosis was appropriate (AID group), whereas in 47 patients (37.0%), the initial diagnosis was inappropriate (IID group).

**Table 1:** Clinical characteristics (n = 127).

Age (years)	69.0±15.4
Male/Female	49/78
Past History	
Hypertension	86 (67.7%)
Diabetes Mellitus	6 (4.7%)
Hyperlipidemia	12 (9.4%)
Ischemic Heart Disease	6 (4.7%)
Cerebrovascular Accident	19 (15.0%)
True aneurysm	2 (1.6%)
Aortic Dissection	4 (3.2%)
Aortic Valvular Disease	9 (7.1%)
Presenting Symptoms	
Pain	82 (64.6%)
Pain not clear	34 (26.7%)
No pain	11 (8.7%)
Sudden onset of pain	64/82 (78.0%)
Disturbed consciousness	51 (40%)
Cold sweat	70 (55.1%)
Initial Vital signs	
BP (mmHg)	105±37.4
HR (bpm)	76.2±20.9
RR (/min)	23.3±5.5
Shock (<90 mmHg)	61 (48.0%)
BP>160 mmHg	13 (10.2%)
Mode of presentation	
Ambulance	100 (78.7%)
Walk in	27 (21.3%)
Any Complications	
Yes	99 (80.0%)
No	28 (20.0%)
Tamponade	45 (35.4%)
Aortic Regurgitation	43 (33.9%)
Peripheral Arterial Occlusion	35 (27.6%)
Coronary Malperfusion	7 (5.5%)
Kidney Ischemia	5 (3.9%)
Visceral Ischemia	4 (3.2%)
Hemiplegia	7 (5.5%)
Paraplegia	1 (0.8%)
Congestive Heart Failure (clinical)	10 (7.9%)
Chest X-p	
Wide Mediastinum	102 (80.3%)
Cardiomegaly	93 (73.2%)
Congestive Heart Failure (radiographic)	22 (17.3%)
Electrocardiogram	
Any abnormalities	98 (77.2%)
Chronic changes	54 (42.5%)
Acute ST-T changes	67 (52.8%)
Normal	29 (22.8%)
Type of dissection	
Classic	90 (70.9%)
IMH	37 (29.1%)

BP: Blood Pressure, HR: Heart Rate (beat per minute), RR: Respiratory Rate (/minute), IMH: intra mural hematoma

The median and mode of TFD were 1.5 and 0.5 h, respectively (first quartile, 0.5 h and third quartile, 4.0 h), and 100 of 127 patients (78.7%) were diagnosed with AAD within 4 h (ED), whereas 27 patients (21.3%) had a TFD longer than 4.5 h (DD).

**Fig 1:**
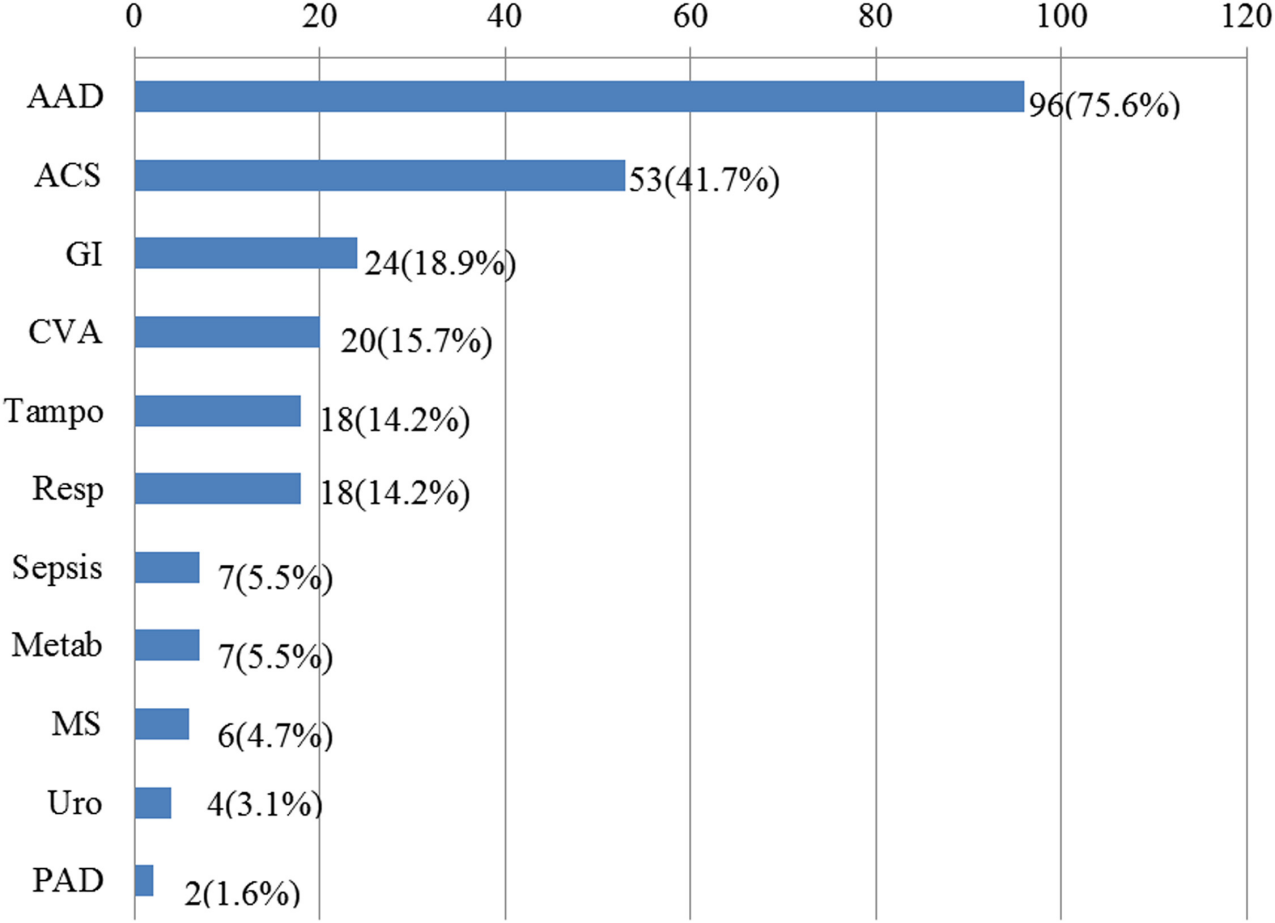
Incidence and distribution of diseases included in the initial differential diagnoses. AAD: acute aortic dissection; ACS: acute coronary syndrome; GI: Gastrointestinal emergencies; Resp: respiratory emergencies; Metab: metabolic disorders; MS: musculoskeletal; Uro: urological emergencies; PAD; peripheral arterial disease. Other abbreviations are as in Tables 2–4.

Fig 1 shows the incidence and distribution of the diseases included in the initial differential diagnoses. AAD was the most common initial working diagnosis, followed by ACS and gastrointestinal diseases, such as gallstones, pancreatitis, and perforated peptic ulcer. CVA, tamponade (of unspecified etiology), and respiratory diseases, such as pneumothorax, pleuritis, and pulmonary emboli, were also frequently included in the initial differential diagnoses. Of note, in over one quarter of patients (36 of 127), AAD was not included in the initial differential diagnoses. These patients comprised the IID group. In addition, patients with inappropriate initial imaging tests were also included in the IID group with a total number of 47 (37%).

Table 2 shows the predictors of IID. Basic clinical characteristics, initial presenting symptoms, distribution of complications, and ECG or chest X-ray findings were not different between the IID and AID groups. WI presentation was significantly more frequent in the IID group. Coronary malperfusion, absence of shock, and classic dissection tended to be associated with IID. After multivariate analysis, WI presentation and coronary malperfusion were significant predictors for IID. Other variables did not exhibit statistically significant results.

**Table 2:** Clinical predictors for inappropriate initial diagnosis.

	AID (n = 80)	IID (n = 47)	univariate P	multivariate P
Age	69.6±14.2	68.0±17.5	0.587	
Male Sex	29 (36.3%)	20 (42.6%)	0.48	
SBP(mmHg)	104±42.7	106±26.4	0.755	
HR	75.0±21.7	78.0±19.6	0.432	
RR	23.0±5.3	23.7±5.8	0.574	
HTN	54(67.5%)	32 (68.1%)	0.95	
DM	3(3.8%)	3(6.4%)	0.81	
HL	8 (10.0%)	4(8.5%)	1.0	
IHD	4(5.0%)	2(4.3%)	1.0	
CVA	11(13.8%)	8((17.0%)	0.62	
TA	1(1.1%)	1(2.1%)	1.0	
DA	3(3.8%)	1(2.1%)	1.0	
AV	5(6.3%)	4(8.5%)	0.9	
WI	12(15.0%)	15(32.0%)	0.024	0.048(2.60:CI = 1.01–6.72)
Pain	53(66.3%)	29(61.7%)	0.605	
SP	41(51.3%)	23(48.9%)	0.801	
MP	9(11.3%)	5(10.6%)	1.0	
Cold sweat	44(55.0%)	26(55.3%	0.972	
DC	36(45.0%)	16(34.0%)	0.225	
Comp	65(81.3%)	34(72.3%)	0.242	
Tampo	31(38.8%)	14(29.8%)	0.308	
AR	27(33.8%)	16(34.0%)	0.973	
PAO	22(27.5%)	13(27.8%)	0.984	
CM	2(2.5%)	5(10.7%)	0.124	0.035(6.48 CI = 1.14–36.8)
HP	4(5.0%)	3(6.4%)	1.0	
CHF (Clinical)	7(8.8%)	3(6.4%)	0.89	
Shock	43(53.8%)	18(38.3%)	0.092	0.266(0.63 CI = 0.28–1.42)
ECG change	64(80%)	34(72.3%)	0.321	
CECG	34(42.5%)	20(42.5%)	0.995	
AECG	44(55.0%)	23(48.9%)	0.509	
MW	64(80.0%)	38(80.9%)	1.0	
Card M	60(75.0%)	33(70.2%)	0.556	
CHF (X-p)	14(17.5%)	8(17.0%)	1.0	
Classic	53(66.3%)	37(78.7%)	0.135	0.286(1.63 CI = 0.67–3.94)
Before 2002	46(57.5%)	31(66.0%)	0.346	

AID: appropriate initial diagnosis; IID: inappropriate initial diagnosis; SBP: systolic blood pressure; HR: heart rate; RR: respiratory rate; HTN: hypertension; HL: hyperlipidemia; DM: diabetes mellitus; IHD: ischemic heart disease; CVA: cerebrovascular accident; TA: true aneurysm; DA: dissection aneurysm; AV: aortic valvular disease; WI: Walk-in visit to the emergency room; SP: sudden pain; MP: migrating pain. Comp: complication; PAO: peripheral arterial occlusion; DC: disturbed consciousness; AR: aortic regurgitation; Tampo: tamponade; CM: coronary malperfusion; HP: hemiplegia; CHF: congestive heart failure; AECG: acute electrocardiagram changes; CECG: chronic ECG changes; MW: mediastinal widening; Car M: cardiomegaly; CHF (X-p): CHF on chest X-p; Classic: classic aortic dissection. Before 2002 indicates the comparison between before 2002 and after 2003.

Table 3 shows the TFD with respect to each clinical variable. The TFD was significantly longer in the IID and WI groups. Although statistically not significant, the TFD tended to be longer in patients with coronary malperfusion and CHF. The TFD was also somewhat longer in groups without any complications, PAO, shock, or consciousness disturbance. Absence of pain in the ER was not associated with a longer TFD.

**Table 3:** Comparison of TFD with respect to clinical variables (n = 127).

	YES	NO	
	Median (Q1-Q3) / n	Median (Q1-Q3 / n	P value
HTN	1.5 (0.5–4.0) / 86	1.0 (0.5–3.0) / 41	0.287
HL	1.5 (1.0–3.5) /12	1.5 (0.5–4.0) / 115	0.555
DM	1.0 (0.5–2.65) / 6	1.5 (0.5–4.0) / 121	0.35
IHD	1.5 (1.0–2.5) / 6	1.5 (0.5–4.0) / 121	0.777
CVA	2.0 (1.0–6.0) / 19	1.0 (0.5–3.0) / 108	0.142
TA	1.75 (0.5–3.0) /2	1.5 (0.5–4.0) / 125	0.742
DA	1.25 (0.625–3.375) / 4	1.5 (0.5–4.0) /123	0.736
AV	1.5 (1.0–12.0) / 9	1.5 (0.5–3.0) / 118	0.248
IID	6.0 (3.0–11.5) / 47	1.0 (0.5–1.5) / 80	<0.001
WI	3.0 (1.0–7.25) / 27	1.0 (0.5–2.75) / 100	0.003
Pain	1.0 (0.5–2.5) /82	2.0 (0.5–4.75) / 45	0.233
Comp	1.5 (0.5–3.0) / 99	2.0 (1.0–6.0) / 28	0.114
Shock	1.0 (0.5–4.0) / 61	1.5 (1.0–3.0) / 66	0.141
PAO	1.5 (0.5–3.0) /35	1.5 (0.625–4.375) / 92	0.179
DC	1.0 (0.5–3.75) / 52	1.5 (1.0–4.0) / 75	0.195
AR	1.5 (1.0–3.0) / 43	1.5 (0.5–4.0) / 84	0.992
Tampo	1.0 (0.5–4.25) / 45	1.5 (1.0–3.0) / 82	0.282
CM	4.0 (1.5–9.0) / 7	1.5 (0.5–3.0) / 120	0.066
HP	1.0 (0.5–3.0) / 7	1.5 (0.5–4.0) / 120	0.618
CHF	2.75 (1.0–21.5) / 10	1.5 (0.5–3.0) / 117	0.106
NECG	1.0 (0.5–4.75) / 29	1.5 (0.5–4.0) / 98	0.891
CECG	1.5 (0.5–3.0) / 54	1.0 (0.5–4.0) / 73	0.747
AECG	1.0 (0.5–4.0) / 67	1.5 (0.5–3.0) / 60	0.473
Car M	1.5 (0.5–4.0) / 93	1.5 (0.875–3.875) / 22	0.814
MW	1.5 (0.5–4.0) / 102	1.0 (0.5–2.75) / 25	0.352
CHF (X-p)	1.75 (0.5–13.0) / 22	1.5 (0.5–3.0) / 105	0.265
Classic	1.5 (0.5–4.0) / 90	1.5 (1.0–3.75) / 37	0.352

Values are expressed as median [first quartile (Q1) − third quartile (Q3)]/n.

NECG: normal ECG, Classic: classic aortic dissection. Other abbreviations are as in Table 2.

Table 4 shows the predictors for DD. The vast majority of the DD group were also in the IID group; i.e., IID was the most powerful predictor for DD. However, the reverse is not true; i.e., IID does not necessarily result in DD. Twenty-two percent of the ED group were in the IID group. Univariate analysis showed that IID and WI were significantly more frequent in the DD group than in the ED group. Absence of PAO and the presence of coronary malperfusion tended to be more frequent in the DD group, although not to a significant extent. Multivariate analysis showed only IID as a predictor for DD. When the analysis was performed after excluding IID as a variable (because IID may naturally result in DD), WI was the only predictor for DD.

**Table 4:** Predictors for DD.

	ED (n = 100)	DD (n = 27)	Univariate P	Multivariate P
Age	68.7±15.5	70.3±15.4	0.62	
Sex (M)	42(42.0%)	7(25.9%)	0.181	
SBP	106±39.0	100±31.0	0.3	
HR	73.9±19.0	85.0±25.2	0.044	
RR	22.9±5.1	24.5±6.7	0.36	
HTN	68(68.0%)	18(66.7%)	1.0	
DM	5(5.0%)	1(3.7%)	1.0	
HL	10(10.0%)	2(7.4%)	1.0	
IHD	6(6.0%)	0(0.0%)	0.34	
CVA	13(13.0%)	6(22.2%)	0.236	
TA	2(2.0%)	0(0%)	1.0	
DA	4(4.0%)	0(0%)	0.578	
AV	5(5.0%)	4(14.8%)	0.095	
IID	22(22.0%)	25(92.6%)	<0.001	<0.001
WI	16(16.0%)	11(40.7%)	0.005	0.107(0.009 if IID not included)
Pain	67(67.0%)	15(55.6%)	0.364	
SP	52(52.0%)	12(44.4%)	0.522	
MP	12(12.0%)	2(7.4%)	0.733	
DC	43(43.0%)	9(33.3%)	0.165	
Comp	80(80.0%)	19(70.4%)	0.302	
Tampo	34(34.0%)	11(40.7%)	0.507	
AR	36(36.0%)	7(25.9%)	0.368	
PAO	31(31.0%)	4(14.3%)	0.144	0.051(0.227 if IID not included)
CM	4(4.0%)	3(11.1%)	0.165	0.831(0.106 if IID not included)
HP	6(6.0%)	1(3.7%)	1.0	
CHF	6(6.0%)	4(14.8%)	0.218	
Shock	52(52.0%)	14(51.9%)	1.0	
AECG	53(53.0%)	14(51.9%)	1.0	
CECG	43(43.0%)	11(40.7%)	1.0	
NECG	22(22.0%)	7(25.9%)	0.796	
MW	79(79.0%)	23(85.1%)	0.592	
Car M	74(74.0%)	19(70.4%)	0.592	
CHF(X-p)	15(15.0%)	7(25.9%)	0.249	
Classic	72(72.0%)	18(66.7%)	0.636	

ED: early diagnosis; DD: delayed diagnosis. Other abbreviations are as in Table 2.

Table 5 shows the comparisons of clinical presentation of WI vs. ambulance transport patients. Patients presenting with shock, disturbed consciousness, and cardiac tamponade were more likely to be transported by ambulance as were those without pain. However, multivariate analysis revealed no single predictor for WI visit.

**Table 5:** Predictors for walk-in visit to the emergency room.

	Walk in (n = 27)	Ambulance (n = 100)	univariate p	multivariate p
Pain	24 (88.9%)	58 (58.0%)	0.003	0.149
Comp	17 (63.0%)	82 (82.0%)	0.064	0.787
Shock	5 (18.5%)	56 (56.0%)	0.001	0.3
PAO	5 (28.5%)	30 (30.0%	0.332	
DC	3 (11.1%)	49 (49.0)	<0.001	0.076
AR	12 (44.4%)	31 (31.0%)	0.252	
Tampo	3 (11.1%)	42 (42.0%)	0.003	0.674
CM	0 (0.0%)	7 (7.0%)	0.344	
HP	1 (1.0%)	6 (6.0%)	1	
CHF	3 (24.0%)	7 (7.0%)	0.442	

Abbreviations are as in Table 2

**Fig 2:**
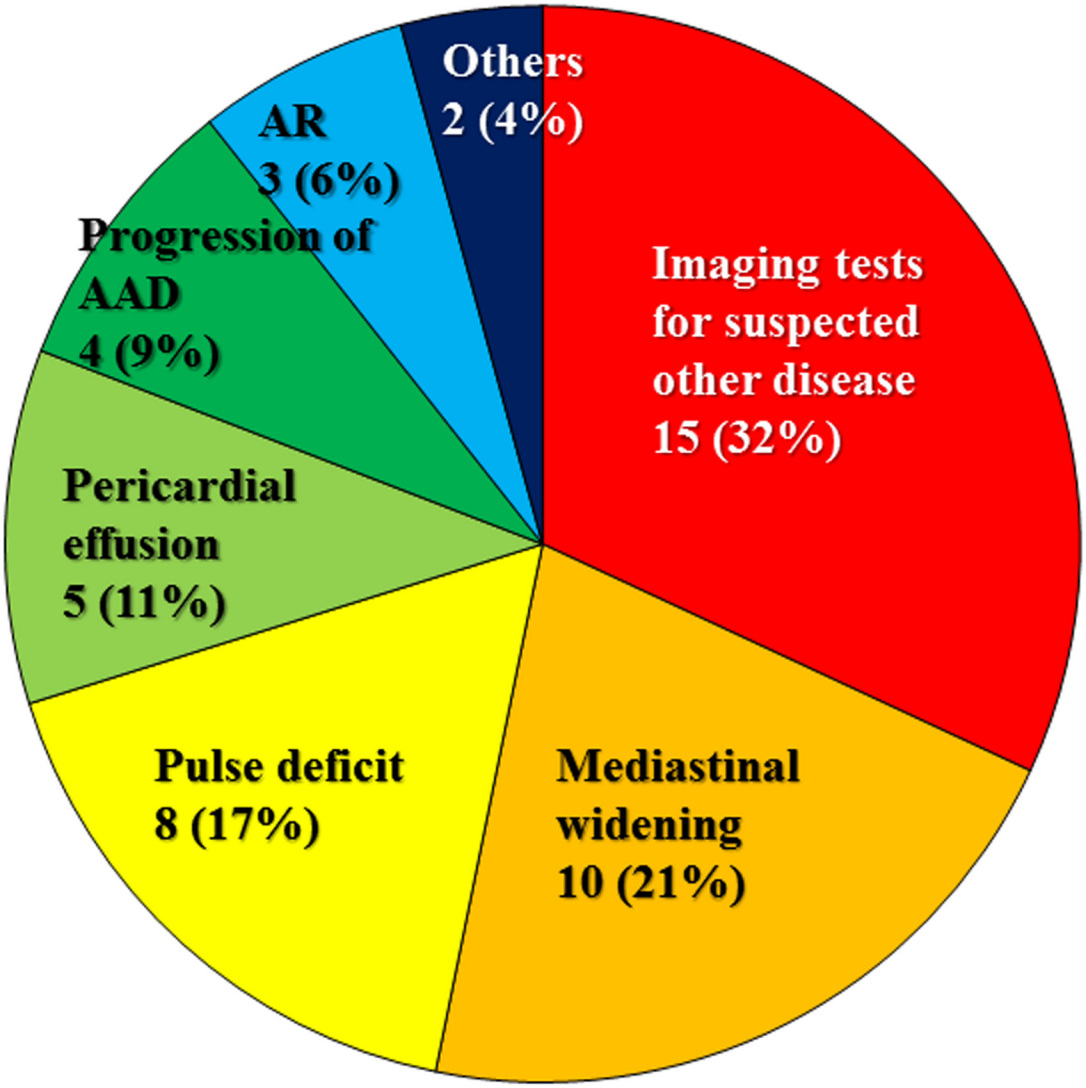
Keys to final diagnosis in the IID group (n = 47). Abbreviations are as in Tables 2–4.

Fig 2 shows the crucial factors to making the correct final diagnosis in patients in the IID group: ordering the tests to evaluate disease processes other than AAD that resulted in unexpected findings of AAD and review and recognition of important findings suggestive of AAD such as widening of the mediastinum on chest X-ray, pulse deficit, or pericardial effusion that have been overlooked initially. Occasionally, the progression of the ongoing dissecting process may lead to the final diagnosis.

## Discussion

The incidence of IID and DD for acute AAD were 37% and 21%, respectively, in the present study. WI and coronary malperfusion were predictors for IID (Table 2), and WI and IID were predictors for DD (Table 4). In approximately a quarter of the patients, AAD was not included in the initial differential diagnoses. In addition, ACS, CVA, and gastrointestinal and pleuropulmonary diseases were frequently included in the initial diagnoses (Fig 1). The incidence of misdiagnosis or DD may vary depending on the definition. Results from this study were comparable to Spittel et al. [5] who reported that 38% of AAD patients had been misdiagnosed on initial evaluation. Kurabayashi et al. [10] reported that the incidence of misdiagnosis was 16% if misdiagnosis was defined as the failure to diagnose AAD at the end of the initial evaluation in the ER. WI mode of presentation, anterior chest pain, and absence of mediastinal widening were risk factors for misdiagnosis. Harris et al. [9] evaluated TFD in the International Registry of Acute Aortic Dissection study and reported a median TFD of 4.3 h (interquartile range of 1.5–24 h). This means that up to one quarter of patients with AAD had a TFD longer than 24 h. Female sex, atypical presentation (no sudden pain or no pain), absence of pulse deficit, or hypotension as well as presentation to a non—tertiary-care hospital were all predictors of DD of AAD. Based on the database of the metropolitan AAD network in Bologna, Italy, Rapezzi et al. [8] reported a median TFD of 177 min and risk factors for diagnostic delay (longer than 12 h, third quartile) including pleural effusion, dyspneic presentation, age <70 years, SBP >105 mmHg, troponin positivity, and an ACS-like ECG.

This study was unique in that predictors of IID (misdiagnosis) and DD were evaluated separately. When the diagnosis of AAD is appropriate, the TFD is significantly shorter. However, the reverse was not true. In the ED group, 22 of 100 patients (22%) had an IID. The key to a final diagnosis in the IID group (Fig 2) shows that occasionally, imaging tests that were not ordered to diagnose AAD (abdominal echography or CT for suspected acute abdomen, for example) lead to an unexpected early diagnosis, meaning that ED is not totally equal to appropriate diagnosis. This observation appeared to be consistent with those of Kurabayashi et al. [10] who reported that the number of imaging tests in patients with correctly diagnosed AAD tended to be higher.

In the present study, the median TFD was much shorter than those previously reported. In our hospital, bedside focused echocardiography and CT have been easily accessible around the clock in the ER since the early 1980s. This may have contributed to the shorter TFD compared with other studies [8,9].

Some of the clinical variables deserve comment. Although the most common presenting symptom was pain in the present study as well as others [1–6], taking a correct history was difficult in the ER in 26.7%, because as many as 40% of the patients had a disturbed consciousness (Table 1). The median TFD tended to be longer in those with disturbed consciousness and those without pain (Table 3), although this was not statistically significant. Nallamothu et al. [12] reported that up to 20% of type A AAD patients had syncope. Patients with tamponade or stroke are more likely to present with syncope. The cause of disturbed consciousness in the present study appeared to be hypotension due to tamponade and CVA (Table 1). The combination of disturbed consciousness and hypotension is potentially important to consider in the diagnosis of AAD, as CVA is usually associated with normal or high blood pressure at presentation to the ER.

Acute ST depression and T wave changes are rather common in the very acute phase of AAD [2,13–15] and are closely related to tamponade and shock rather than mechanical coronary malperfusion [13]. In the present study, acute ST depression and T wave changes were not a significant predictor for IID or DD. In contrast, coronary malperfusion is a significant predictor of IID. If there are profound ECG changes suggestive of coronary malperfusion, such as ST elevation in inferior leads or diffuse ST depression and ST elevation of aVr [15–18], then misdiagnosis of classic acute myocardial infarction due to occlusion of the right coronary artery or left main trunk may be made instead of AAD, subjecting the patient to inappropriate therapy [19–22]. These observations are consistent with those of Rapezzi et al. [8], who noted that an ACS-like ECG was a risk for DD. The only means of avoiding unnecessary and possibly harmful cardiac catheterization is to perform quick bedside focused echocardiography to find the intimal flap in the aorta or pericardial effusion [23,24]. If there is further suspicion of AAD, then chest CT with contrast enhancement should be performed before proceeding with cardiac catheterization.

Patients who present with CHF (clinical) tended to have longer TFD in the present study. This was consistent with observations by Rapezzi et al. [8] that dyspnea as a symptom was a predictor of DD, i.e., if the physician is focused on CHF owing to symptoms of dyspnea, then the diagnosis of AAD may be missed.

Absence of shock (or hypotension, i.e., SBP <105 mmHg) was a predictor for DD as noted by Rapezzi et al. [8] and Harris et al.[9]. In the present study, patients with shock tended to have shorter TFD. These findings indicate that making a diagnosis of shock early may lead to earlier diagnostic imaging tests, which in turn may lead to earlier diagnosis of AAD.

PAO is an important sign of AAD, and if present, it significantly increases the likelihood of AAD [25]. Absence of PAO was a risk factor for DD in the study of Harris et al [9]. In the present study, absence of PAO was a borderline significant predictor for DD.

The mode of presentation was an important predictor of DD. Harris et al. [9] noted that not being taken directly to the tertiary care hospital was a significant predictor for DD. The present study as well as that of Kurabayashi et al. [10] show that WI visit to the ER was a significant predictor for IID and DD. Unfortunately, there was no single predictor for WI visit using multivariate analysis.

### Limitations

The present study was a retrospective study in a single institution over a wide time range and was based only on the information easily available in the ER. Only those who presented directly to our ER within 12 h from symptom onset were included, which may explain the apparently sicker patient population in our study than that in other studies. We are not aware of any reasonable explanation of the higher proportion of female patients in our study population.

## Conclusion

Inappropriate or delayed diagnosis of AAD is still relatively common. What were considered to be classic symptoms and findings, such as pain, are often absent, and clinical manifestations are diverse. This wide variety of clinical manifestations itself is typical of AAD. If a patient with AAD presents to the ER with symptoms mimicking other diseases, such as ACS, CHF, or CVA, then a correct diagnosis may be missed and delayed. A high index of suspicion and prompt imaging tests appear to be most important in the ER.
